# Validation of a derived version of the IPF-specific Saint George’s Respiratory Questionnaire

**DOI:** 10.1186/s12931-021-01853-2

**Published:** 2021-10-05

**Authors:** Thomas Skovhus Prior, Nils Hoyer, Saher Burhan Shaker, Jesper Rømhild Davidsen, Ole Hilberg, Haridarshan Patel, Elisabeth Bendstrup

**Affiliations:** 1 Center for Rare Lung Diseases, Department of Respiratory Diseases and Allergy, Aarhus University Hospital, Aarhus, Denmark; 2 Department of Respiratory Medicine, Herlev-Gentofte University Hospital, Copenhagen, Denmark; 3 South Danish Center for Interstitial Lung Diseases (SCILS), Department of Respiratory Medicine, Odense University Hospital, Odense, Denmark; 4 Department of Respiratory Medicine, Vejle Hospital, Vejle, Denmark; 5 Immensity Consulting, Inc., Chicago, IL USA

**Keywords:** Idiopathic pulmonary fibrosis, Quality of life, Validation, St. George’s Respiratory Questionnaire, IPF-specific version of St. George’s Respiratory Questionnaire

## Abstract

**Background:**

Health-related quality of life (HRQL) is impaired in patients with idiopathic pulmonary fibrosis (IPF). HRQL is often measured using the St. George’s Respiratory Questionnaire (SGRQ) despite the development of an IPF-specific version (SGRQ-I). Using data from a real-world cohort of patients with IPF, we aimed to transform SGRQ into a derived version of SGRQ-I, SGRQ-I_der_, to examine the cross-sectional and longitudinal validity of SGRQ-I_der_ and to compare SGRQ-I_der_ to SGRQ-I.

**Methods:**

Based on results from SGRQ, SGRQ-I_der_ was derived applying the algorithm used to develop SGRQ-I. Of the 50 items in SGRQ, 34 items were retained in SGRQ-I_der_. Response options for seven items were collapsed and minor adjustments were made to the weights of two items after correspondence with the developers of SGRQ-I. Cross-sectional validation, responsiveness and minimal clinically important difference (MCID) were assessed by comparison to other HRQL instruments, pulmonary function tests and 6-min walk test performed at baseline, 6 and 12 months. Furthermore, the association between SGRQ-I_der_ scores and mortality was examined.

**Results:**

A total of 150 IPF patients participated and 124 completed follow-up at 12 months. SGRQ-I_der_ performed comparably to SGRQ-I with a high concurrent validity, good test–retest reliability and high known-groups validity. SGRQ-I_der_ was responsive to change in HRQL and physiological anchors. MCID of SGRQ-I_der_ for improvement and deterioration was 3.5 and 5.7, respectively. SGRQ-I_der_ scores were associated with mortality in both univariate (HR 1.82, 95% CI 1.42–2.34 per 20-point increase) and multivariate analyses (HR 1.57, 95% CI 1.20–2.05 per 20-point increase).

**Conclusions:**

The SGRQ-I_der_ is a valid, reliable and responsive HRQL instrument in patients with IPF and has psychometric properties comparable to SGRQ-I. Thus, SGRQ results can reliably be transformed into the SGRQ-I_der_. The MCID estimates were calculated for improvement and deterioration separately. Increasing SGRQ-I_der_ score was associated with increased mortality.

**Supplementary Information:**

The online version contains supplementary material available at 10.1186/s12931-021-01853-2.

## Introduction

Idiopathic pulmonary fibrosis (IPF) is a chronic fibrotic lung disease with a large respiratory symptom burden and poor prognosis [1]. Dyspnea, cough, fatigue, social isolation, loss of emotional well-being and numerous comorbidities lead to impaired health-related quality of life (HRQL) in patients with IPF [2–5]. As HRQL is regarded as an increasingly important outcome, both in clinical trials and daily clinical practice, valid and reliable HRQL measures are required [6].

Various instruments are used to measure HRQL in patients with IPF. Due to the lack of IPF-specific HRQL instruments, the St. George’s Respiratory Questionnaire (SGRQ) has often been used in IPF studies even though it was developed for patients with obstructive lung diseases [7, 8]. SGRQ has adequate psychometric properties in IPF, but patients with obstructive lung diseases have a different symptom profile, and some items in SGRQ are less relevant to patients with IPF. Hence, especially the symptoms domain of SGRQ has weaker psychometric properties in IPF [8].

To meet these drawbacks, an IPF-specific version of SGRQ (SGRQ-I) was developed and validated [5, 9, 10]. Only the items most relevant to patients with IPF were retained, resulting in a 34-item instrument compared to the 50 items in SGRQ. However, SGRQ-I has not been widely adopted and SGRQ is still used broadly in clinical trials. The assessment of HRQL could probably be more specific to patients with IPF, if the results from clinical trials using SGRQ, instead of SGRQ-I, could be transformed into an equally valid and reliable derived version of SGRQ-I (SGRQ-I_der_). The SGRQ-I_der_ should be validated both cross-sectionally and longitudinally to ensure solid psychometric properties.

Using data from a real-world cohort of patients with IPF, the aim of this study was to transform SGRQ into a derived version of SGRQ-I (SGRQ-I_der_), to examine the cross-sectional and longitudinal validity of SGRQ-I_der_ and to compare SGRQ-I_der_ to SGRQ-I. Furthermore, we aimed to determine the minimal clinically important difference (MCID) for SGRQ-I_der_ and to examine the ability of SGRQ-I_der_ to predict mortality in patients with IPF.

## Materials and methods

### Study subjects

The current study was based on the cohort previously used for cross-sectional and longitudinal validation of SGRQ-I and the King’s Brief Interstitial Lung Disease questionnaire (K-BILD) as well as comorbidities in IPF [4, 5, 10, 11]. Adult patients with a guideline-based diagnosis of IPF and attending one of the tertiary interstitial lung disease (ILD) centers in Denmark at the university hospitals in Aarhus, Copenhagen and Odense were eligible for inclusion [18, 19]. Inability to complete the instruments due to linguistic or cognitive barriers excluded patients from participation.

### Study measures

Based on results from SGRQ, SGRQ-I_der_ was derived applying the algorithm used to develop SGRQ-I [9]. The derivation algorithm was determined by two separate authors (TSP and HP) and compared; in case of disagreement, consensus was obtained in consultation with EB. Of the 50 items in SGRQ, the 34 items most relevant to patients with IPF were retained in SGRQ-I_der_. Response options for seven items were collapsed (all items in the symptoms domain and the first item in the activities domain, Additional file 1: Table S1). Minor adjustments were made to the weights of two items compared to the SGRQ-I algorithm after correspondence with the developers of SGRQ-I, Jeff Swigris and Janelle Yorke (reverse scoring in the last item in the symptoms domain and a minor correction to the weight of the first item in the activities domain, Additional file 1: Table S2).

At baseline, SGRQ was completed along with SGRQ-I, K-BILD, Short Form-36 (SF-36) and University of California San Diego Shortness of Breath questionnaire (SOBQ). Global Rating of Change Scales (GRCS) and SGRQ were completed 14 days later. Patients completed GRCS, SGRQ, SGRQ-I, K-BILD, and SOBQ after 6 and 12 months.

Pulmonary function tests (PFT) were performed to assess forced vital capacity (FVC) and diffusing capacity of the lung for carbon monoxide (DLCO) along with a 6-min walk test (6MWT) at baseline, 6 and 12 months. Based on this information, the gender, age and physiology (GAP) index was calculated.

Comorbidities were registered at baseline by review of patients’ medical history, indications for medications, blood samples, echocardiography and chest high-resolution computed tomography (HRCT) scans, and the Charlson comorbidity index was calculated.

*SGRQ-I* contains 34 items measuring HRQL and has been cross-sectionally and longitudinally validated for use in IPF [5, 9, 10]. SGRQ-I scores were divided into deteriorated (ΔSGRQ-I ≥ 4.9), unchanged (ΔSGRQ-I − 3.9 to 4.9) and improved (ΔSGRQ-I ≤ − 3.9) in accordance with the MCIDs for SGRQ-I [10].

*K-BILD* is a HRQL instrument developed for patients with ILD and validated in IPF [4, 10, 12]. Based on the MCIDs for K-BILD, patients were classified as deteriorated (ΔK-BILD ≤ − 2.7), unchanged (ΔK-BILD − 2.7 to 4.7) and improved (ΔK-BILD ≥ 4.7) [10].

*SOBQ* measures dyspnea related to daily activities [13]. SOBQ has both cross-sectional and longitudinal validity in IPF [14]. In accordance with the MCID of SOBQ in IPF, changes in SOBQ score were classified as deteriorated (ΔSOBQ ≥ 8), unchanged (ΔSOBQ − 8 to 8) or improved (ΔSOBQ ≤ − 8) [14].

*SF-36* is a generic instrument assessing a range of quality of life domains [15].

*GRCS* ranging from − 5 “Very much worse” over 0 “Unchanged” to 5 “Very much better” were used to estimate changes from baseline in HRQL [16]. Four specific GRCS were composed: one to reflect the overall HRQL and three for the domains of SGRQ-I_der_. Results from each GRCS was categorized as deteriorated (− 5 to − 2), unchanged (− 1 to 1) or improved (2 to 5).

*FVC* and *DLCO* are commonly used in IPF as indicators of disease severity, and functional capacity can be evaluated by the distance walked during the 6-min walk test (*6MWD*). Mortality in patients with IPF is associated with both PFTs and 6MWD [17, 18]. Based on the MCID for FVC in ILD, patients with an absolute change in FVC % predicted below 6% were regarded as unchanged, whereas an absolute change larger than or equal to ± 6% was regarded as improved or deteriorated, respectively [19, 20].

As no MCID has been reported for DLCO in IPF, the intraindividual variability in DLCO measurements was used [21]. Absolute changes in DLCO % predicted were divided into deteriorated (ΔDLCO ≤ − 10%), unchanged (ΔDLCO − 10% to 10%) and improved (ΔDLCO ≥ 10). Changes in 6MWD were divided into deteriorated (Δ6MWD ≤ − 28 m), unchanged (Δ6MWD − 28 m to 28 m) and improved (Δ6MWD ≥ 28 m), in concordance with the MCID for 6MWD in IPF [22].

*The GAP index* was developed to predict mortality in patients with IPF [23]. The index is calculated based on gender, age, FVC and DLCO. The resulting three groups have 1-year mortalities ranging from 6 to 39%.

### Statistical analyses

Instruments with more than 15% missing answers or missing either total or domain scores were excluded from the analyses.

#### Cross-sectional validation

Baseline results were used to perform the cross-sectional validation of SGRQ-I_der_. Floor and ceiling effects were defined by > 15% of patients scoring the highest or lowest possible scores, respectively. Difference between SGRQ-I and SGRQ-I_der_ total and domain scores were assessed by the paired two sample t-test.

The internal consistency measures the interrelatedness of items in an instrument. To examine the internal consistency of SGRQ-I_der_, Cronbach’s α was calculated for the total and each domain score. Results > 0.7 were compatible with a reliable internal consistency [24].

Concurrent validity was evaluated by comparing SGRQ-I_der_ to SGRQ-I, K-BILD, SOBQ, SF-36, PFTs and 6MWD. Intraclass correlation coefficients (ICCs) (2,1) and Bland–Altman plots were used to compare SGRQ-I_der_ to SGRQ-I, and SGRQ-I_der_ was compared to the other measures using Pearson’s correlation coefficients.

By comparing the scores at baseline and after two weeks in stable patients (scoring − 1 to 1 in GRCS 2 weeks after baseline), test–retest reliability of SGRQ-I_der_ was evaluated by ICCs (2,1) and a Bland–Altman plot. ICCs > 0.7 were regarded as a measure of reliability [24].

Known-groups validity was assessed by evaluating the ability of SGRQ-I_der_ to differentiate between patients with different stages of disease severity. Patients were divided into “known groups” of disease severity according to 6MWD and PFTs (upper and lower quartiles), use of long-term oxygen therapy (LTOT) and GAP index [25]. The independent two-sample t-test was used for normally distributed continuous data and the Wilcoxon–Mann–Whitney test for not normally distributed continuous data. GAP groups were compared by linear regression analyses.

Data were analyzed using STATA, version 14.2 (StataCorp, College Station, Texas).

#### Longitudinal validation

Change in SGRQ-I_der_ total score was analyzed using a mixed effects model with cluster effect for center (using the “Clustered Sandwich Estimator”) and random intercept.

Responsiveness was assessed using Pearson’s correlation coefficients to examine the association between changes in SGRQ-I_der_ and changes in anchors (GRCS, SGRQ-I, K-BILD, SOBQ, FVC % predicted, DLCO % predicted, 6MWD) from baseline to 12 months. Negative correlations between SGRQ-I_der_ and GRCS, K-BILD, FVC, DLCO and 6MWD were expected because of inverse scoring algorithms. Subgroup analyses to evaluate the effect of receiving antifibrotic treatment at baseline were performed.

The association between SGRQ-I_der_ baseline score stratified into 20-point intervals and mortality for up to 48 months of follow-up was assessed using Cox regression analyses. Subsequently, the model was adjusted for age, FVC % predicted and the Charlson comorbidity index.

The MCID SGRQ-I_der_ was estimated by receiver operating characteristic (ROC) curves. To estimate MCID, both anchor-based and distribution-based methods are recommended [26, 27], and a combination of these methods are included in ROC curves [28]. A correlation coefficient > 0.3 between SGRQ-I_der_ and anchors to be included in the MCID analyses was prespecified, as anchors and the instrument under investigation must be related [26]. Based on thresholds of the anchors (described above), patients were categorized as deteriorated, unchanged or improved. Separate ROC curves were used to estimate the MCID for deterioration (unchanged vs. deteriorated patients) and improvement (unchanged vs. improved patients) [28]. The optimal cut-off point of the ROC curve (with equal sensitivity and specificity) was regarded as the MCID estimate for each anchor. To assess the influence of antifibrotic therapy at baseline on MCID estimates, subgroup analyses were performed by similar ROC curve analyses.

## Results

The study population consisted of 150 patients with IPF included from August 2016 to March 2018 (Table 1). The cohort was dominated by male patients receiving antifibrotic therapy at baseline with a history of smoking. DLCO was moderately reduced whereas FVC was relatively well preserved. Most patients completed the 6-month visit (n = 135, 90%) and the 12-month visit (n = 124, 83%). Patients were withdrawn from the study due to death (n = 16), inability to complete the instruments (n = 1), inability to attend the outpatient clinic (n = 3) or patient’s wish to withdraw (n = 6).

**Table 1:** Baseline characteristics

Characteristics	Value
Total cohort, n	150
Male gender (%)	122 (81.3%)
Age, years (SD)	72.9 ± 6.2
Smoking status	
Never (%)	40 (26.6%)
Former (%)	101 (67.3%)
Current (%)	9 (6.0%)
FVC, % predicted (SD)	87.2 ± 23.1
DLCO, % predicted (SD)	48.4 ± 14.1
6MWD, m (SD)	450.3 ± 112.5
Concurrent long-term oxygen therapy (%)	19 (12.7%)
Antifibrotic treatment, n (%)	85 (56.7%)
Pirfenidone, n (%)	51 (34.0%)
Nintedanib, n (%)	34 (22.7%)
Charlson comorbidity index (IQR)	1 (0–2)

Values are presented as *n* (%), mean ± standard deviation (SD), or median with interquartile range (IQR) [4, 5, 10, 11]

FVC: Forced vital capacity; DLCO: Diffusing capacity of the lung for carbon monoxide; 6MWD: distance walked during the 6-min walk test

### Cross-sectional validation

No floor or ceiling effects in SGRQ-I_der_ total or domain scores were present. Only minor differences in item, total and domain scores between SGRQ-I_der_ and SGRQ-I were observed (Table 2 and Additional file 1: Table S3). A good internal consistency was indicated by high Cronbach’s α results in both domain and total scores (Table 2).

**Table 2:** Summary scores, internal consistency and concurrent validity of SGRQ-I_der_

SGRQ-I_der_	SGRQ-I_der_ Mean (SD)	SGRQ-I Mean (SD)	Difference (95% CI)	Cronbach’s α	ICC
Total	43.3 (21.5)	43.4 (22.1)	0.03 (− 0.45 to 0.51)	0.92	0.99
Symptoms	49.8 (22.9)	49.6 (27.1)	− 0.10 (− 2.74 to 2.53)	0.74	0.80
Activities	61.5 (28.3)	61.5 (28.3)	0.03 (0.02 to 0.03)	0.86	1.00
Impacts	32.0 (22.3)	31.9 (22.3)	0.12 (− 0.07 to 0.31)	0.84	1.00

Data are presented as mean with standard deviations (SD) or 95% confidence interval (CI) of SGRQ-I_der_ compared to SGRQ-I and Cronbach’s alpha and intraclass correlation coefficients (ICC) of SGRQ-I_der_ for the total and three domain scores in all patients

SGRQ-I_der_: IPF-specific version of the St. George’s Respiratory Questionnaire derived from results from the original St. George’s Respiratory Questionnaire

**Fig. 1:**
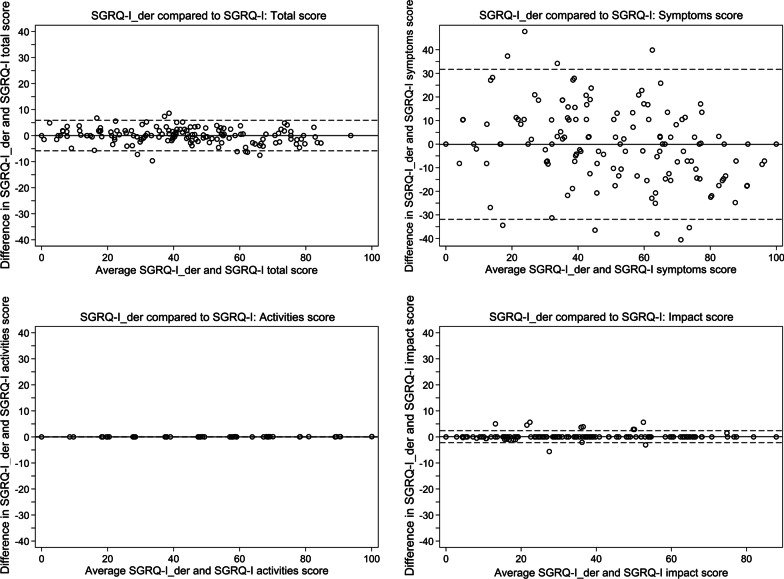
Bland–Altman plot of the agreement between SGRQ-I_der_ and SGRQ-I. The solid line represents the mean difference and dashed lines represent 95% limits of agreement. SGRQ-I_der_: IPF-specific version of the St. George’s Respiratory Questionnaire derived from results from the original St. George’s Respiratory Questionnaire; SGRQ-I: IPF-specific version of the St. George’s Respiratory Questionnaire

A high concurrent validity was demonstrated by high ICCs and Bland–Altman plots comparing SGRQ-I_der_ to SGRQ-I (Table 2 and Fig. 1). These results were supported by moderate to strong correlations with K-BILD, SOBQ and SF-36 and weaker correlations with PFTs and 6MWD (Table 3 and Additional file 1: Table S4).

**Table 3:** Concurrent validity of SGRQ-I_der_

	SGRQ-I_der_ total	SGRQ-I_der_ symptoms	SGRQ-I_der_ activities	SGRQ-I_der_ impacts
K-BILD total	− 0.76	− 0.57	− 0.71	− 0.70
K-BILD chest symptoms	− 0.68	− 0.58	− 0.54	− 0.67
K-BILD breathlessness and activities	− 0.78	− 0.58	− 0.76	− 0.70
K-BILD psychological	− 0.58	− 0.44	− 0.52	− 0.55
SOBQ total	0.80	0.52	0.73	0.77
SF-36 PCS	− 0.71	− 0.50	− 0.63	− 0.69
SF-36 MCS	− 0.46	− 0.40	− 0.34	− 0.46
FVC % predicted	− 0.29	− 0.23	− 0.20	− 0.30
DLCO % predicted	− 0.49	− 0.31	− 0.53	− 0.43
6MWD (m)	− 0.51	− 0.26	− 0.47	− 0.51

Data are presented as Pearson’s correlation coefficients for all patients

SGRQ-I_der_: IPF-specific version of the St. George’s Respiratory Questionnaire derived from results from the original St. George’s Respiratory Questionnaire; SGRQ-I: IPF-specific version of the St. George’s Respiratory Questionnaire; K-BILD: King’s Brief Interstitial Lung Disease questionnaire; SOBQ: University of California, San Diego Shortness of Breath Questionnaire; SF-36: Short Form-36; PCS: Physical Component Score; MCS: Mental Component Score; FVC: Forced vital capacity; DLCO: Diffusing capacity of the lung for carbon monoxide; 6MWD: Distance walked during the 6-min walk test

**Fig. 2:**
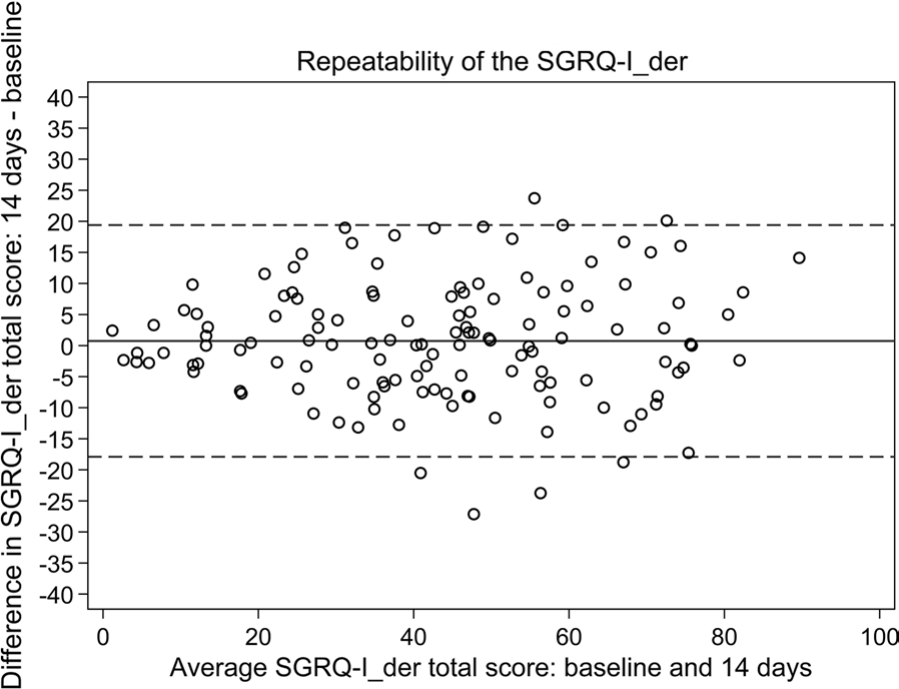
Bland–Altman plot of the test–retest reliability of SGRQ-I_der_ in all stable patients. The solid line represents the mean difference and dashed lines represent 95% limits of agreement. SGRQ-I_der_: IPF-specific version of the St. George’s Respiratory Questionnaire derived from results from the original St. George’s Respiratory Questionnaire

Most patients remained in a stable health status after 14 days as indicated by GRCS. Based on results from the stable patients, high ICCs and a Bland–Altman plot indicated a good test–retest reliability of SGRQ-I_der_, which was comparable to SGRQ-I (Table 4, Fig. 2 and Additional file 1: Table S5).

**Table 4:** Test–retest reliability of SGRQ-I_der_

SGRQ-I_der_	*n*	ICC
Total	99 (73.9%)	0.91
Symptoms	105 (78.4%)	0.77
Activities	104 (77.6%)	0.80
Impacts	104 (77.6%)	0.76

Data represent number of stable patients (% of responders, *n* = 134) and intraclass correlation coefficients (ICCs) [5]

SGRQ-I_der_: IPF-specific version of the St. George’s Respiratory Questionnaire derived from results from the original St. George’s Respiratory Questionnaire

**Fig. 3:**
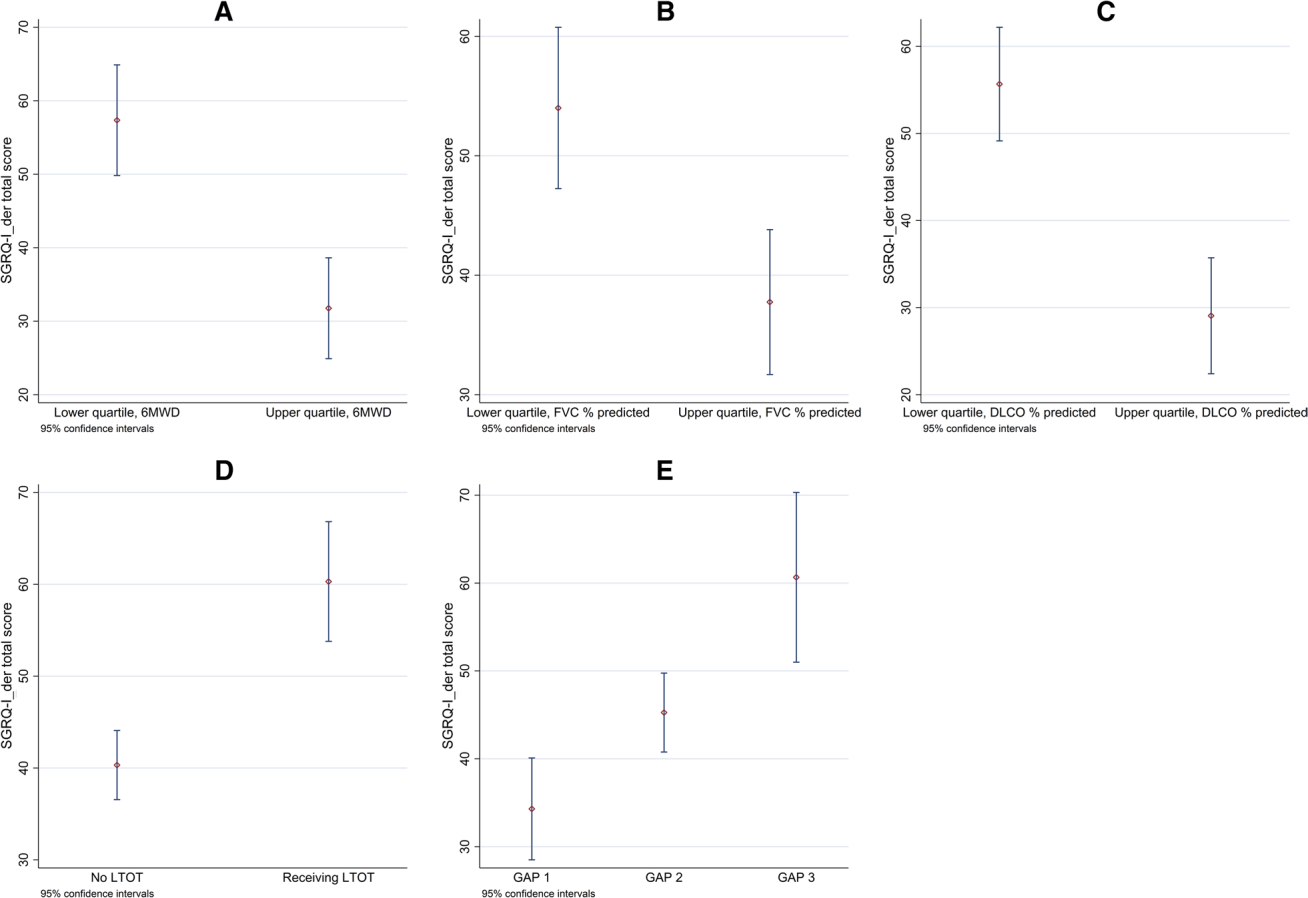
SGRQ-I_der_ total score (mean and 95% confidence intervals) in **A** the lower and upper quartile of 6MWD, **B** the lower and upper quartile of FVC % predicted, **C** the lower and upper quartile of DLCO % predicted, **D** LTOT, and **E** GAP index. SGRQ-I_der_: IPF-specific version of the St. George’s Respiratory Questionnaire derived from results from the original St. George’s Respiratory Questionnaire; 6MWD: distance walked during the 6-min walk test; FVC: Forced vital capacity; DLCO: Diffusing capacity of the lung for carbon monoxide; LTOT: Long-term oxygen therapy; GAP: Gender, age and physiology index

The known-groups validity was high, as SGRQ-I_der_ demonstrated significantly better HRQL in patients with the highest quartiles of PFTs and 6MWD compared with the lowest quartiles. Better HRQL was also seen in patients without LTOT and decreasing HRQL was associated with increasing disease severity as indicated by the GAP index (Fig. 3).

### Longitudinal validation

After 12 months, a non-significant HRQL decrease was observed (ΔSGRQ-I_der_ total: 2.7, 95% CI − 0.4 to 7.8). According to the HRQL anchors, 16–33% of patients improved and 20–44% deteriorated, whereas 2–20% improved and 16–35% deteriorated according to the physiological anchors.

SGRQ-I_der_ was responsive to changes in HRQL anchors and, to a lesser extent, also physiological anchors similar to SGRQ-I (Table 5 and Additional file 1: Table S6). Correlations for patients receiving antifibrotic therapy at baseline were comparable. All correlations were in the expected direction.

**Table 5:** Correlations between changes in SGRQ-I_der_ domains and changes in anchors from baseline to 12 months

	GRCS	ΔSGRQ-I	ΔK-BILD	ΔSOBQ	ΔFVC%	ΔDLCO%	Δ6MWD
ΔSGRQ-I_der_ total	− 0.55	0.95	− 0.56	0.61	− 0.24	− 0.24	− 0.48
ΔSGRQ-I_der_ symptoms	− 0.51	0.62	− 0.45	0.46	− 0.27	− 0.23	− 0.38
ΔSGRQ-I_der_ activities	− 0.47	0.68	− 0.48	0.44	− 0.23	− 0.21	− 0.29
ΔSGRQ-I_der_ impacts	− 0.46	0.87	− 0.44	0.53	− 0.13	− 0.17	− 0.45

Δ: Change from baseline to 12 months; SGRQ-I_der_: IPF-specific version of the St. George’s Respiratory Questionnaire derived from results from the original St. George’s Respiratory Questionnaire; GRCS: Global rating of change scales; SGRQ-I: IPF-specific version of the St. George’s Respiratory Questionnaire; K-BILD: King’s Brief Interstitial Lung Disease questionnaire; SOBQ: University of California San Diego Shortness of Breath questionnaire; FVC%: Forced vital capacity % predicted; DLCO%: Diffusing capacity of the lung for carbon monoxide % predicted; 6MWD: Distance walked during the 6-min walk test

The MCID estimates for SGRQ-I_der_ are presented in Table 6. Subgroup analyses for patients receiving antifibrotic treatment at baseline were comparable (SGRQ-I_der_ total score: improvement 3.9, deterioration 6.5).

**Table 6:** Mean and range of MCID estimates for SGRQ-I_der_ domains based on change in anchors from baseline to 12 months

Domains	Improvement		Deterioration	
	Mean	Range	Mean	Range
SGRQ-I_der_ total	3.5	2.9–4.5	5.7	4.7–6.6
SGRQ-I_der_ symptoms	7.9	7.5–8.2	3.9	1.7–5.3
SGRQ-I_der_ activities	10.2	10.0–10.9	9.6	9.5–9.6
SGRQ-I_der_ impacts	1.2	0.3–2.8	4.1	2.3–5.8

MCID: Minimal clinically important difference; SGRQ-I_der_: IPF-specific version of the St. George’s Respiratory Questionnaire derived from results from the original St. George’s Respiratory Questionnaire

**Fig. 4:**
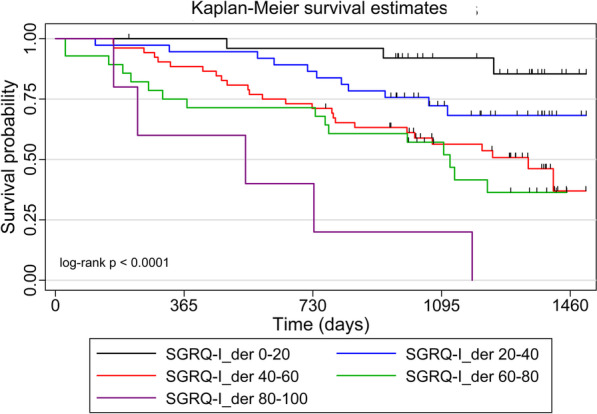
Survival estimates for groups of patients with different SGRQ-I_der_ total score. SGRQ-I_der_: IPF-specific version of the St. George’s Respiratory Questionnaire derived from results from the original St. George’s Respiratory Questionnaire

Increasing baseline SGRQ-I_der_ total score in 20-point intervals was associated with increasing mortality in the univariate model (HR 1.82, 95% CI 1.42–2.34) and remained significant after adjustment for FVC % predicted, age and the Charlson comorbidity index (HR 1.57, 95% CI 1.20–2.05) (Fig. 4).

## Discussion

This is the first study to directly transform SGRQ results from a real-world, multicenter cohort of patients with IPF into a derived version of SGRQ-I and to evaluate the validity and reliability of this HRQL instrument. Given the extensive use of SGRQ and limited utilization of SGRQ-I in IPF research, results may become suboptimal due to the content and psychometric properties of SGRQ being less specific for patients with IPF. Our study showed that SGRQ data can be transformed into SGRQ-I_der_ with a validity, reliability and responsiveness comparable to SGRQ-I in patients with IPF. The mean total and domain scores of SGRQ-I_der_ were almost identical to the mean scores of SGRQ-I, and as such performed comparably to SGRQ-I with only minor differences. MCID estimates for improvement and deterioration were estimated, facilitating the interpretation of repeated measurements. Furthermore, SGRQ-I_der_ can be used to assess the prognosis of IPF.

The internal consistency of SGRQ-I_der_ was high across both total score and the three domains, but the symptoms domain had the lowest performance. The same pattern was observed concerning concurrent validity. Generally, the symptoms domain had weaker correlations with both HRQL instruments and physiological parameters and in the Bland–Altman plots, the variation between SGRQ-I and SGRQ-I_der_ exceeded the variation in the other domains. These differences could partly be explained by the changes made to the instrument during the development of SGRQ-I. In the symptoms domain, two items from SGRQ were removed, the response options in the remaining items were collapsed, and new weights were calculated for each response options. In the other domains, selected items were removed and response options and weights were mostly unchanged [9]. As the largest changes were made in the symptoms domain, the major differences between SGRQ-I and SGRQ-I_der_ would be expected in this domain. Another explanation could be found in the inherent properties of the symptoms domain. The domain includes items concerning sputum, wheezing and attacks of chest trouble which are less relevant to patients with IPF. Therefore, the symptoms domain also has the weakest psychometric properties compared with the other domains of SGRQ and SGRQ-I when used in IPF populations [5, 8]. Despite these shortcomings, most items in the symptoms domain were preserved when developing the SGRQ-I, as the validity and reliability of the domain became weaker without the items and at the same time, the instrument would be better at assessing HRQL in IPF patients with comorbidities such as chronic obstructive pulmonary disease [9].

MCID estimates for SGRQ-I_der_ were comparable to MCIDs for SGRQ-I (improvement 3.5 vs. 3.9 and deterioration 5.7 vs. 4.9, respectively) [10]. The small differences are probably caused by the minor deviations that exist between the two versions of the instrument. MCID estimates for SGRQ in other IPF studies were slightly higher ranging from 4 to 6.6 [25, 29]. The divergence could be caused by a single MCID for deterioration and improvement combined, by characteristics of the cohorts and the statistical methods used. The SGRQ studies were based on clinical trial populations, whereas the present study was based on a real-world cohort of patients. Furthermore, more distribution-based approaches were used in these studies which tends to produce higher MCID estimates [30]. When using ROC curves, both anchor- and distribution-based methods are included in the model as recommended [28].

There was a clear association between decreasing baseline HRQL and increasing mortality, even after adjustment for covariates. The relationship between HRQL and mortality in IPF has been studied using SGRQ, but results are divergent. In two studies, baseline HRQL was shown to be significantly associated with mortality [31, 32], whereas two other studies did not find a significant association in multivariate analyses [33, 34]. Kreuter et al. found a significant association between HRQL at last available follow-up and mortality, but no association with change in HRQL from baseline [35]. Hence, it is possible that the IPF-specific versions of SGRQ are superior at predicting mortality due to the disease-specific nature of the instruments.

A strength of the present study is the inclusion of a large and broad, multicenter, real-world cohort of patients with IPF based on wide inclusion and few exclusion criteria. This increases the external validity of the results. Furthermore, the other HRQL instruments used for the analyses were validated for use in IPF, thus increasing the reliability of the results. A limitation is the possible recall bias related to GRCS used in the longitudinal analyses. Even though it can be difficult to recall ones health status 12 months ago, GRCS provide a simple evaluation of patients’ HRQL and can be tailored to reflect both overall HRQL and domains of a HRQL instrument. In addition, GCRS have good validity, reliability and response to change over time [16]. Another potential limitation is healthy volunteer bias, as healthier patients may be more willing to participate in clinical trials [36]. This may limit the generalizability of the results, but the current cohort also included patients with advanced disease, thus limiting this type of bias.

In conclusion, a derived version of SGRQ-I transformed from SGRQ data, the SGRQ-I_der_, is a valid, reliable and responsive HRQL instrument in patients with IPF and has psychometric properties comparable to SGRQ-I. Thus, SGRQ results can reliably be transformed into the SGRQ-I_der_. The MCID estimate for improvement is 3.5 and 5.7 for deterioration, and increasing SGRQ-I_der_ scores are associated with increased mortality.

## Supplementary Information

**Additional file 1:Table S1.** The SGRQ-I development algorithm. **Table S2.** Changes from the original scoring algorithm for SGRQ-I used in SGRQ-I_der_. **Table S3.** Mean (SD) item scores at baseline. **Table S4.** Concurrent validity of SGRQ-I_der_ and SGRQ-I. **Table S5.** Test-retest validity of SGRQ-I_der_ and SGRQ-I. **Table S6.** Responsiveness of SGRQ-I_der_ and SGRQ-I

## Data Availability

The datasets collected and analyzed during the current study are not publicly available due to information that could compromise research participants’ privacy, but are available from the corresponding author on reasonable request.
