# Supplementary material for: Validation of a derived version of the IPF-specific Saint George’s Respiratory Questionnaire
Source: Respir Res. 2021 Oct 5;22:259. doi: 10.1186/s12931-021-01853-2 (PMC8491388; doi:10.1186/s12931-021-01853-2)
Supplement: Supplementary file 1 — Additional file 1:Table S1. The SGRQ-I development algorithm. Table S2. Changes from the original scoring algorithm for SGRQ-I used in SGRQ-Ider. Table S3. Mean (SD) item scores at baseline. Table S4. Concurrent validity of SGRQ-Ider and SGRQ-I. Table S5. Test-retest validity of SGRQ-Ider and SGRQ-I. Table S6. Responsiveness of SGRQ-Ider and SGRQ-I [file 12931_2021_1853_MOESM1_ESM.docx]

**Validation of the Saint George’s Respiratory Questionnaire, IPF-specific version derived from the original Saint George’s Respiratory Questionnaire**

Thomas Skovhus Prior, Nils Hoyer, Saher Burhan Shaker, Jesper Rømhild Davidsen, Ole Hilberg, Haridarshan Patel, Elisabeth Bendstrup

**Additional file**

**Table S1.** The SGRQ-I development algorithm

| **Domain** | **SGRQ** | | **SGRQ weight** | **SGRQ-I weight** | **SGRQ-I** | |
| --- | --- | --- | --- | --- | --- | --- |
| **Symptoms** | **Item 1: coughed:** | **Most of the days** | **80.6** | **80.6** | **Almost everyday** | **Item 1: I have coughed:** |
|  |  | **Several days a week** | **63.2** | **40.2** | **Only with Infection** |  |
|  |  | **A few days a month** | **29.3** |  |  |  |
|  |  | **Only with Infection** | **28.1** |  |  |  |
|  |  | **Not at all** | **0** | **0** | **Not at all** |  |
|  | **Item 2: brought up phlegm** | **Most of the days** | **76.8** | **76.8** | **Almost everyday** | **Item 2: brought up phlegm** |
|  |  | **Several days a week** | **60** | **41.4** | **Only with Infection** |  |
|  |  | **A few days a month** | **34** |  |  |  |
|  |  | **Only with Infection** | **30.2** |  |  |  |
|  |  | **Not at all** | **0** | **0** | **Not at all** |  |
|  | **Item 3: shortness of breadth** | **Most of the days** | **87.2** | **87.2** | **Almost everyday** | **Item 3: shortness of breadth** |
|  |  | **Several days a week** | **71.4** | **50.3** | **Only with Infection** |  |
|  |  | **A few days a month** | **43.7** |  |  |  |
|  |  | **Only with Infection** | **35.7** |  |  |  |
|  |  | **Not at all** | **0** | **0** | **Not at all** |  |
|  | **Item 4: wheezing** | **Most of the days** | **86.2** | **86.2** | **Almost everyday** | **Item 4: wheezing** |
|  |  | **Several days a week** | **71** | **51** | **Only with Infection** |  |
|  |  | **A few days a month** | **45.6** |  |  |  |
|  |  | **Only with Infection** | **36.4** |  |  |  |
|  |  | **Not at all** | **0** | **0** | **Not at all** |  |
|  | **Item 5: attackes of chest trouble** | **More than 3** | **86.7** | **66.2** | **More than 1** | **Item 5: attackes of chest trouble** |
|  |  | **3 attacks** | **73.5** |  |  |  |
|  |  | **2 attacks** | **60.3** |  |  |  |
|  |  | **1 attack** | **44.2** |  |  |  |
|  |  | **No attacks** | **0** | **0** | **None** |  |
|  | **Item 6: length of worst attack** |  |  | **DELETED** | | |
|  | **Item 7: how many good days** | **No good days** | **93.3** | **0** | **None** | **Item 6: how many good days** |
|  |  | **1 or 2 days** | **76.6** | **51.2** | **A few days** |  |
|  |  | **3 or 4 days** | **61.5** |  |  |  |
|  |  | **nearly every day** | **15.4** |  |  |  |
|  |  | **every day** | **0** | **93.3** | **Every day** |  |
|  | **Item 8: wheeze worst in the morning** |  |  | **DELETED** | | |
| **Impacts** | **Item 9: Importance of chest condition** |  |  | **DELETED** | | |
|  | **Item 10: paid employment** | **Stop work** | **88.9** | **83.3** | **Interferes or stop** | **Item 1: paid employment** |
|  |  | **Interferes or changed** | **77.6** |  |  |  |
|  |  | **Not affected** | **0** | **0** | **Not affected** |  |
|  | **Item 12a: cough hurts** | **true** | **81.1** | **81.1** | **true** | **Item 2: cough hurts** |
|  |  | **false** | **0** | **0** | **false** |  |
|  | **Item 12b: cough makes me tired** | **true** | **79.1** | **79.1** | **true** | **Item 3: cough makes me tired** |
|  |  | **false** | **0** | **0** | **false** |  |
|  | **Item 12c: breathless when I talk** | **true** | **84.5** | **84.5** | **true** | **Item 4: breathless when I talk** |
|  |  | **false** | **0** | **0** | **false** |  |
|  | **Item 12d: breathless when I bend over** | **true** | **76.8** | **76.8** | **true** | **Item 5: breathless when I bend over** |
|  |  | **false** | **0** | **0** | **false** |  |
|  | **Item 12e: disturbs my sleep** | **true** | **87.9** | **87.9** | **true** | **Item 6: disturbs my sleep** |
|  |  | **false** | **0** | **0** | **false** |  |
|  | **Item 12f: exhausted easily** | **true** | **84** | **84** | **true** | **Item 7: exhausted easily** |
|  |  | **false** | **0** | **0** | **false** |  |
|  | **Item 13a: embarrassing in public** | **true** | **74.1** | **74.1** | **true** | **Item 8: embarrassing in public** |
|  |  | **false** | **0** | **0** | **false** |  |
|  | **Item 13b: nuisance to family, …** | **true** | **79.1** | **79.1** | **true** | **Item 9: nuisance to family, …** |
|  |  | **false** | **0** | **0** | **false** |  |
|  | **Item 13c: panic or afraid** | **true** | **87.7** | **87.7** | **true** | **Item 10: panic or afraid** |
|  |  | **false** | **0** | **0** | **false** |  |
|  | **Item 13d: Not in control** | **true** | **90.1** | **90.1** | **true** | **Item 11: Not in control** |
|  |  | **false** | **0** | **0** | **false** |  |
|  | **Item 13e: chest not getting better** |  |  | **DELETED** | | |
|  | **Item 13f: become frail or invalid** |  |  | **DELETED** | | |
|  | **Item 13g: exercise not safe** | **true** | **75.7** | **75.7** | **true** | **Item 12: exercise not safe** |
|  |  | **false** | **0** | **0** | **false** |  |
|  | **Item 13h: everything too much effort** | **true** | **84.5** | **84.5** | **true** | **Item 13: everything too much effort** |
|  |  | **false** | **0** | **0** | **false** |  |
|  | **Item 14a: medication does not help** |  |  | **DELETED** | | |
|  | **Item 14b: embarrassed using medication** |  |  | **DELETED** | | |
|  | **Item 14c: unpleasant side effects** |  |  | **DELETED** | | |
|  | **Item 14d: medication interferes with life** |  |  | **DELETED** | | |
|  | **Item 16a: cannot play sports or games** | **true** | **64.8** | **64.8** | **true** | **Item 14: cannot play sports or games** |
|  |  | **false** | **0** | **0** | **false** |  |
|  | **Item 16b: entertainment** |  |  | **DELETED** | | |
|  | **Item 16c: shopping** | **true** | **81** | **81** | **true** | **Item 15: shopping** |
|  |  | **false** | **0** | **0** | **false** |  |
|  | **Item 16d: housework** | **true** | **79.1** | **79.1** | **true** | **Item 16: housework** |
|  |  | **false** | **0** | **0** | **false** |  |
|  | **Item 16e: far from bed or chair** | **true** | **94** | **94** | **true** | **Item 17: far from bed or chair** |
|  |  | **false** | **0** | **0** | **false** |  |
|  | **Item 17: how chest affects you** | **does not stop** | **0** | **0** | **does not stop** | **Item 18: how chest affects you** |
|  |  | **stop 1 or 2 things** | **42** | **42** | **stop 1 or 2 things** |  |
|  |  | **stop most things** | **84.2** | **84.2** | **stop most things** |  |
|  |  | **stops everything** | **96.7** | **96.7** | **stops everything** |  |
| **Activities** | **Item 11a: Sitting or lying** |  |  | **DELETED** | | |
|  | **Item 11b: washing or dressing** | **true** | **82.8** | **82.2** | **true** | **Item 1: washing or dressing** |
|  |  | **false** | **0** | **0** | **false** |  |
|  | **Item 11c: walking around home** | **true** | **80.2** | **80.2** | **true** | **Item 2: walking around home** |
|  |  | **false** | **0** | **0** | **false** |  |
|  | **Item 11d: walking outside on level** | **true** | **81.4** | **81.4** | **true** | **Item 3: walking outside on level** |
|  |  | **false** | **0** | **0** | **false** |  |
|  | **Item 11e: walking up flight of stairs** | **true** | **76.1** | **76.1** | **true** | **Item 4: walking up flight of stairs** |
|  |  | **false** | **0** | **0** | **false** |  |
|  | **Item 11f: walking up hills** |  |  | **DELETED** | | |
|  | **Item 11g: playing sports or games** | **true** | **72.1** | **72.1** | **true** | **Item 5: playing sports or games** |
|  |  | **false** | **0** | **0** | **false** |  |
|  | **Item 15a: long for washing or dressing** |  |  | **DELETED** | | |
|  | **Item 15b: cannot take bath** |  |  | **DELETED** | | |
|  | **Item 15c: walk more slowly** |  |  | **DELETED** | | |
|  | **Item 15d: housework take long time** | **true** | **70.6** | **70.6** | **true** | **Item 6: housework take long time** |
|  |  | **false** | **0** | **0** | **false** |  |
|  | **Item 15e: walking up stairs, go slowly** | **true** | **71.6** | **71.6** | **true** | **Item 7: walking up stairs, go slowly** |
|  |  | **false** | **0** | **0** | **false** |  |
|  | **Item 15f: if I hurry, have to slow down** | **true** | **72.3** | **72.3** | **true** | **Item 8: if I hurry, have to slow down** |
|  |  | **false** | **0** | **0** | **false** |  |
|  | **Item 15g: breathing makes difficult to walk up hills** | **true** | **74.5** | **74.5** | **true** | **Item 9: breathing makes difficult to walk up hills** |
|  |  | **false** | **0** | **0** | **false** |  |
|  | **Item 15h: breathing makes difficult to carry heavy loads** |  |  | **DELETED** | | |
|  | **Item 15i: breathing makes difficult heavy manual work** | **true** | **63.5** | **63.5** | **true** | **Item 10: breathing makes difficult heavy manual work** |
|  |  | **false** | **0** | **0** | **false** |  |

*SGRQ-I*: IPF-specific version of the St. George’s Respiratory Questionnaire.

**Table S2.** Changes from the original scoring algorithm for SGRQ-I used in SGRQ-I_der_

| **Item** | **Responses** | **Original scoring** | **Revised scoring** |
| --- | --- | --- | --- |
| SGRQ-I Symptoms item 6:  How many good days | None | 0 | 93.3 |
|  | A few days | 51.2 | 51.2 |
|  | Every day | 93.3 | 0 |
| SGRQ-I Activities item 1:  Washing or dressing | True | 82.2 | 82.8 |
|  | False | 0 | 0 |

*SGRQ-I*: IPF-specific version of the St. George’s Respiratory Questionnaire, *SGRQ-I_der_*: IPF-specific version of the St. George’s Respiratory Questionnaire derived from results from the original St. George’s Respiratory Questionnaire

**Table S3.** Mean (SD) item scores at baseline

| **Items** | **SGRQ-I_der_** | **SGRQ-I** |
| --- | --- | --- |
| **S1** | 1.57 (0.65) | 1.52 (0.76) |
| **S2** | 1.91 (0.76) | 1.77 (0.86) |
| **S3** | 1.90 (0.73) | 1.75 (0.87) |
| **S4** | 2.57 (0.64) | 2.45 (0.79) |
| **S5** | 1.62 (0.49) | 1.51 (0.50) |
| **S6** | 2.14 (0.53) | 2.59 (0.61) |
| **A1** | 0.33 (0.47) | 0.33 (0.47) |
| **A2** | 0.18 (0.38) | 0.18 (0.38) |
| **A3** | 0.42 (0.50) | 0.42 (0.50) |
| **A4** | 0.68 (0.47) | 0.68 (0.47) |
| **A5** | 0.78 (0.42) | 0.78 (0.42) |
| **A6** | 0.63 (0.49) | 0.63 (0.49) |
| **A7** | 0.64 (0.48) | 0.64 (0.48) |
| **A8** | 0.81 (0.39) | 0.81 (0.39) |
| **A9** | 0.80 (0.40) | 0.80 (0.40) |
| **A10** | 0.92 (0.27) | 0.92 (0.27) |
| **I1** | 1.62 (0.49) | 1.62 (0.49) |
| **I2** | 0.18 (0.38) | 0.18 (0.38) |
| **I3** | 0.42 (0.50) | 0.42 (0.50) |
| **I4** | 0.32 (0.47) | 0.32 (0.47) |
| **I5** | 0.35 (0.48) | 0.35 (0.48) |
| **I6** | 0.23 (0.42) | 0.23 (0.42) |
| **I7** | 0.66 (0.48) | 0.66 (0.48) |
| **I8** | 0.26 (0.44) | 0.26 (0.44) |
| **I9** | 0.21 (0.41) | 0.21 (0.41) |
| **I10** | 0.14 (0.34) | 0.14 (0.34) |
| **I11** | 0.37 (0.49) | 0.37 (0.49) |
| **I12** | 0.61 (0.49) | 0.61 (0.49) |
| **I13** | 0.29 (0.46) | 0.29 (0.46) |
| **I14** | 0.70 (0.46) | 0.70 (0.46) |
| **I15** | 0.09 (0.29) | 0.09 (0.29) |
| **I16** | 0.22 (0.42) | 0.22 (0.42) |
| **I17** | 0.05 (0.21) | 0.05 (0.21) |
| **I18** | 1.86 (0.84) | 1.86 (0.84) |

*SGRQ-I_der_*: IPF-specific version of the St. George’s Respiratory Questionnaire derived from results from the original St. George’s Respiratory Questionnaire, *SGRQ-I*: IPF-specific version of the St. George’s Respiratory Questionnaire.

**Table S4.** Concurrent validity of SGRQ-I_der_ and SGRQ-I

|  | Total | | Symptoms | | Activities | | Impacts | |
| --- | --- | --- | --- | --- | --- | --- | --- | --- |
|  | SGRQ-I_der_ | SGRQ-I | SGRQ-I_der_ | SGRQ-I | SGRQ-I_der_ | SGRQ-I | SGRQ-I_der_ | SGRQ-I |
| K-BILD total | -0.76 | -0.76 | -0.57 | -0.58 | -0.71 | -0.71 | -0.70 | -0.70 |
| K-BILD chest symptoms | -0.68 | -0.69 | -0.58 | -0.64 | -0.54 | -0.54 | -0.67 | -0.66 |
| K-BILD breathlessness and activities | -0.78 | -0.78 | -0.58 | -0.57 | -0.76 | -0.76 | -0.70 | -0.70 |
| K-BILD psychological | -0.58 | -0.58 | -0.44 | -0.47 | -0.52 | -0.52 | -0.55 | -0.55 |
| SOBQ total | 0.80 | 0.80 | 0.52 | 0.54 | 0.73 | 0.74 | 0.77 | 0.76 |
| SF-36 PCS | -0.71 | -0.71 | -0.50 | -0.50 | -0.63 | -0.63 | -0.69 | -0.68 |
| SF-36 MCS | -0.46 | -0.46 | -0.40 | -0.40 | -0.34 | -0.34 | -0.46 | -0.46 |
| FVC% | -0.29 | -0.30 | -0.23 | -0.32 | -0.20 | -0.20 | -0.30 | -0.30 |
| DLCO% | -0.49 | -0.48 | -0.31 | -0.28 | -0.53 | -0.53 | -0.43 | -0.42 |
| 6MWD (m) | -0.51 | -0.50 | -0.26 | -0.25 | -0.47 | -0.46 | -0.51 | -0.52 |

*SGRQ-I_der_*: IPF-specific version of the St. George’s Respiratory Questionnaire derived from results from the original St. George’s Respiratory Questionnaire; *SGRQ-I*: IPF-specific version of the St. George’s Respiratory Questionnaire; *K-BILD*: King’s Brief Interstitial Lung Disease questionnaire; *SOBQ*: University of California, San Diego Shortness of Breath Questionnaire; *SF-36*: Short Form-36; *PCS*: Physical Component Score; *MCS*: Mental Component Score; *FVC*: Forced vital capacity; *DLCO*: Diffusing capacity of the lung for carbon monoxide; *6MWD*: Distance walked during the 6-minute walk test. [E1]

**Table S5.** Test-retest validity of SGRQ-I_der_ and SGRQ-I

| **Domain** | **Stable patients** | **SGRQ-I_der_** | **SGRQ-I** |
| --- | --- | --- | --- |
|  | ***n*** | **ICC** | **ICC** |
| Total | 99 (73.9%) | 0.91 | 0.92 |
| Symptoms | 105 (78.4%) | 0.77 | 0.81 |
| Activities | 104 (77.6%) | 0.80 | 0.80 |
| Impacts | 104 (77.6%) | 0.76 | 0.79 |

Data represent number of stable patients (% of responders, n = 134) and intraclass correlation coefficients (ICCs). *SGRQ-I_der_*: IPF-specific version of the St. George’s Respiratory Questionnaire derived from results from the original St. George’s Respiratory Questionnaire. *SGRQ-I*: IPF-specific version of the St. George’s Respiratory Questionnaire. [E1]

**Table S6.** Responsiveness of SGRQ-I_der_ and SGRQ-I

|  |  | **GRCS** | **ΔSOBQ** | **ΔFVC%** | **ΔDLCO%** | **Δ6MWD** |
| --- | --- | --- | --- | --- | --- | --- |
| **Total** | **ΔSGRQ-I_der_** | -0.55 | 0.61 | -0.24 | -0.24 | -0.48 |
|  | **ΔSGRQ-I** | -0.57 | 0.60 | -0.21 | -0.17 | -0.49 |
| **Symptoms** | **ΔSGRQ-I_der_** | -0.51 | 0.46 | -0.27 | -0.23 | -0.38 |
|  | **ΔSGRQ-I** | -0.44 | 0.39 | -0.25 | -0.05 | -0.33 |
| **Activities** | **ΔSGRQ-I_der_** | -0.47 | 0.44 | -0.23 | -0.21 | -0.29 |
|  | **ΔSGRQ-I** | -0.47 | 0.44 | -0.21 | -0.18 | -0.29 |
| **Impacts** | **ΔSGRQ-I_der_** | -0.46 | 0.53 | -0.13 | -0.17 | -0.45 |
|  | **ΔSGRQ-I** | -0.46 | 0.53 | -0.09 | -0.15 | -0.47 |

Δ: Change from baseline to 12 months, *SGRQ-I_der_*: IPF-specific version of the St. George’s Respiratory Questionnaire derived from results from the original St. George’s Respiratory Questionnaire, *SGRQ-I*: IPF-specific version of the St. George’s Respiratory Questionnaire, *GRCS*: Global rating of change scales, *K-BILD*: King’s Brief Interstitial Lung Disease questionnaire, *SOBQ*: University of California San Diego Shortness of Breath questionnaire, *FVC%*: Forced vital capacity % predicted, *DLCO%*: Diffusing capacity of the lung for carbon monoxide % predicted, *6MWD*: Distance walked during the 6-minute walk test. [E2]

**Bibliography**

E1. Prior TS, Hoyer N, Shaker SB, Davidsen JR, Yorke J, Hilberg O, et al. Validation of the IPF-specific version of St. George’s Respiratory Questionnaire. Respir Res [Internet]. 2019;20:199. Available from: https://doi.org/10.1186/s12931-019-1169-9

E2. Prior TS, Hoyer N, Hilberg O, Shaker SB, Davidsen JR, Bendstrup E. Responsiveness and minimal clinically important difference of SGRQ-I and K-BILD in idiopathic pulmonary fibrosis. Respir Res [Internet]. 2020 [cited 2020 Apr 22];21:91. Available from: https://respiratory-research.biomedcentral.com/articles/10.1186/s12931-020-01359-3
